# Advances in biomechanical modeling of lumbar spine diseases and tumors: gaps, opportunities, and AI integration

**DOI:** 10.3389/fbioe.2026.1786433

**Published:** 2026-06-18

**Authors:** Elizabeth Beaulieu, Marshall Daffner, Dominic Obraitis, Ridge Weston, Samip Patel, Zachary Comella, Maohua Lin, Debojit Biswas, Rudy Paul, Gui Pires, Talha S. Cheema, Frank D. Vrionis

**Affiliations:** 1 Florida Atlantic University Charles E Schmidt College of Medicine, Boca Raton, FL, United States; 2 Biomedical Engineering, Florida Atlantic University, Boca Raton, FL, United States; 3 SurgenTec LLC, Boca Raton, FL, United States; 4 Boca Raton Regional Hospital Inc., Boca Raton, FL, United States

**Keywords:** artificial intelligence, finite element analysis, lumbar spine disorders, musculoskeletal modeling, patient-specific modeling, physics-informed neural networks, spinal biomechanics, surgical planning

## Abstract

Lumbar spine disorders encompass degenerative, deformity, osteoporotic, and neoplastic conditions that alter spinal biomechanics and contribute to pain, instability, and functional impairment. Computational modeling, particularly finite element analysis (FEA), enables estimation of internal stress distributions and load transmission patterns that cannot be directly measured *in vivo*. Although traditional FEA has provided important mechanistic insight, clinical applicability has been limited by labor-intensive model generation, simplified loading assumptions, and reliance on generalized anatomical properties. Recent advances in artificial intelligence (AI) and musculoskeletal modeling have expanded the scalability and physiological realism of computational spine simulation. Automated image segmentation allows efficient generation of patient-specific geometries, while physics-informed neural networks, multibody dynamics, and coupled multibody-finite element frameworks integrate data-driven learning with biomechanical principles to represent functional loading conditions. These approaches support applications including deformity correction planning, fracture risk assessment, implant optimization, and prediction of postoperative mechanical complications. Despite substantial methodological progress, routine clinical translation remains limited. Key challenges include insufficient outcome-based validation, lack of standardized reporting frameworks, and uncertainty regarding generalizability across heterogeneous patient populations. This narrative review synthesizes contemporary computational modeling strategies applied to lumbar spine disease and evaluates their clinical applications, validation requirements, and translational barriers. Emphasis is placed on the evolution of spinal simulation from investigational biomechanics toward patient-specific decision support, while highlighting the validation and outcome-based evidence required for responsible clinical integration.

## 1 Introduction

Lumbar spine disorders, including degenerative conditions and primary or metastatic tumors, represent a leading cause of disability worldwide. Common pathologies such as lumbar spinal stenosis, lumbar disc herniation, degenerative disc disease (DDD), and facet joint arthropathy alter the structural and material properties of spinal tissues, while tumors and osteoporotic weakening compromise vertebral integrity (Katz et al., 2022; El Melhat et al., 2024; Inoue and Espinoza Orías, 2011; Knezevic et al., 2021; Missenard et al., 2020). Although clinically heterogeneous, these conditions share a central biomechanical consequence: disruption of physiological load transmission, motion patterns, and structural stability within the lumbar motion segment (Wang H. et al., 2023).

Understanding the mechanical implications of lumbar pathology is critical for improving surgical planning, implant design, and risk prediction. However, direct *in vivo* quantification of internal spinal stresses, strain distributions, and disc pressures remains limited (Kim et al., 2013). Computational modeling therefore provides a framework for estimating these internal mechanical variables under controlled assumptions.

### 1.1 Experimental and clinical observations

Degenerative processes alter disc composition through dehydration, proteoglycan depletion, collagen remodeling, and annular fissuring, resulting in modified stiffness and altered load-sharing behavior (Inoue and Espinoza Orías, 2011). Clinical and experimental observations demonstrate that degeneration can influence segmental motion patterns and adjacent-level mechanics (Wang H. et al., 2023; Li et al., 2011). Facet joint arthropathy involves osteoarthritic remodeling of the zygapophyseal joints, including cartilage degradation, osteophyte formation, and subchondral changes (Knezevic et al., 2021; Netzer et al., 2018; Acosta Julbe et al., 2024). Disc and facet joint degeneration are biomechanically interdependent, with alterations in one structure influencing loading patterns in the other.

Lumbar spinal stenosis involves narrowing of the spinal canal or neural foramina, often associated with degenerative remodeling of posterior elements (Katz et al., 2022). These structural changes modify posterior load distribution and may contribute to altered segmental kinematics (Ma et al., 2025). Lumbar disc herniation reflects disruption of annular integrity and displacement of disc material, which changes internal disc mechanics and nerve root loading (El Melhat et al., 2024; Palanca et al., 2021).

Tumors of the lumbar spine introduce focal reductions in bone strength and heterogeneous material properties, increasing strain concentrations and fracture risk (Missenard et al., 2020; Palanca et al., 2021; Ahmadi et al., 2025a). Experimental evidence demonstrates that tumor-affected vertebrae exhibit significantly elevated strain compared to healthy bone under mechanical loading (Missenard et al., 2020; Lai et al., 2025).

Although biomechanical alterations are implicated in symptom development and instability (Knezevic et al., 2021; Palanca et al., 2020), internal mechanical variables such as stress and strain distributions cannot be directly measured *in vivo*.

### 1.2 Insights from computational and numerical modeling

Numerical investigations, including finite element analysis (FEA) and related computational modeling approaches, have been used to further characterize the biomechanical interactions between intervertebral discs and facet joints (Park et al., 2024; Bashkuev et al., 2020). These studies demonstrate how variations in disc integrity and facet joint morphology influence internal stress distributions and load-sharing patterns that cannot be directly measured experimentally.

Computational approaches have also been applied to a range of lumbar spine pathologies beyond degeneration, including deformity correction (Imai et al., 2025) and fracture risk assessment (Yan et al., 2022). Alignment disorders such as adult spinal deformity alter global load distribution across multiple spinal segments, while osteoporotic bone loss reduces vertebral strength and modifies failure patterns (Imai et al., 2025; Yan et al., 2022). Incorporating these distinct biomechanical environments highlights the broader applicability of computational simulation across heterogeneous spinal conditions.

Accordingly, computational modeling provides a framework for quantifying these internal mechanical variables under controlled assumptions.

### 1.3 Finite element modeling in lumbar spine biomechanics

FEA enables quantitative simulation of spinal mechanics under controlled boundary and loading conditions. Lumbar FEA models can incorporate nonlinear material behavior, anisotropy of the annulus fibrosus, biphasic disc properties, and contact interactions between facet joints (Cachot et al., 2025). These models allow estimation of internal stress distributions, nucleus pulposus pressure, and segmental motion patterns that are not accessible through clinical imaging alone (Kim et al., 2013).

FEA has been applied to investigate degenerative conditions and tumors, including modeling of disc degeneration, facet loading, and tumor-related instability (Ma et al., 2025; Lai et al., 2025; Park et al., 2024; Bashkuev et al., 2020; Cachot et al., 2025; Zhao et al., 2025). For example, Ma et al. developed a high-fidelity, CT-derived three-dimensional finite element model of the L3-S1 lumbar spine to quantify how disc degeneration interacts with factors such as body mass index and osteoporosis following decompression procedures (Ma et al., 2025). Their simulations revealed that increased degeneration combined with reduced bone quality and elevated BMI produced marked increases in intradiscal pressure and facet-joint stresses under physiological loading. This illustrates how degenerative changes can alter load distribution and internal stresses even when gross range of motion appears modest (Ma et al., 2025). In the context of neoplastic disease, Palanca et al. showed that type, size, and position of metastatic lesions significantly affect vertebral deformation under complex loading conditions, with lytic lesions producing substantially greater strain concentrations compared to healthy controls (Palanca et al., 2021). This work highlighted that incorporating tumor morphology into spine FE models enhances the biomechanical understanding of tumor-induced instability, supporting the use of computational approaches for fracture risk assessment and surgical planning. However, FEA outputs represent model-derived predictions and must be interpreted distinctly from clinical outcomes. For example, although biomechanical comparisons between lumbar fusion and artificial disc replacement have been performed computationally (Zhao et al., 2025), long-term clinical evidence indicates broadly comparable outcomes between these procedures in appropriately selected patients. Therefore, computational findings should be considered complementary to, not substitutes for, clinical data.

Despite its utility, traditional FEA workflows face several limitations. Model generation often requires labor-intensive manual segmentation of imaging data. Many studies rely on generalized or averaged material properties rather than patient-specific inputs. Sensitivity analyses have further demonstrated that geometric variability can substantially influence predicted intradiscal pressure, range of motion, and facet loading, limiting generalizability of fixed-geometry models. Additionally, nonlinear high-fidelity simulations can be computationally expensive, limiting scalability and clinical integration.

Computational modeling of spinal biomechanics has evolved over several decades with early finite element models demonstrating the feasibility of estimating internal load transmission and segmental motion under controlled boundary conditions. Subsequent studies expanded these approaches to incorporate nonlinear material properties, facet contact interactions, and subject-specific geometries across both healthy and pathological lumbar conditions (Garavelli et al., 2022; Vanaclocha-Saiz et al., 2022; Azadi and Arjmand, 2021; Lipscomb et al., 2017; Paholpak et al., 2021). Collectively, these studies established FEA as a central investigative framework for characterizing spinal mechanics and informing experimental and clinical research. As a result, computational modeling has shifted from exploratory simulation toward hypothesis-testing methodology capable of complementing experimental biomechanics.

### 1.4 Artificial intelligence in computational spine modeling

Recent advances in artificial intelligence (AI), particularly deep learning-based image segmentation, are transforming computational biomechanics. Automated segmentation of vertebrae, intervertebral discs, and associated structures from CT and MRI datasets improves reproducibility and reduces processing time. This enables more efficient generation of patient-specific geometries for FEA modeling.

Beyond segmentation, AI techniques have been explored for parameter estimation, probabilistic modeling of degeneration states, and surrogate modeling approaches designed to accelerate simulation while preserving biomechanical fidelity. The integration of AI with finite element modeling (AI-FEA) therefore represents a methodological advancement aimed at improving personalization, scalability, and translational feasibility. In parallel, hybrid multibody-finite element (MB-FE) frameworks have emerged to integrate muscle-driven dynamics with tissue-level stress analysis, offering a more physiologically comprehensive modeling paradigm.

Among emerging AI architectures, physics-informed neural networks (PINNs) incorporate governing mechanical equations directly into neural network training. By embedding physical constraints into loss functions, PINNs aim to preserve mechanical consistency while reducing dependence on large labeled data sets (Zhang et al., 2025). These frameworks have been applied to nonlinear constitutive modeling (Haghighat et al., 2023) and stress-strain relationship discovery from limited experimental data (Niu et al., 2023). Although largely developmental, such hybrid approaches illustrate ongoing efforts to bridge data-driven learning with mechanistic modeling in spinal biomechanics.

Despite methodological progress, clinical implementation requires rigorous validation, standardized reporting, and careful differentiation between predictive simulation and empirical outcome data. Without such safeguards, there is risk of overinterpretation and inappropriate clinical extrapolation.

### 1.5 Scope and objectives

This narrative review examines lumbar spine pathologies from a computational biomechanics’ perspective, including degeneration, deformity, fracture, and tumor-related instability. Specifically, we:

1. Summarize current FEA modeling approaches applied to degenerative and neoplastic lumbar conditions.
2. Evaluate the integration of AI techniques into model development and simulation workflows.
3. Critically assess validation frameworks, translational barriers, hybrid modeling strategies, and requirements necessary for safe and effective clinical implementation.

By synthesizing developments in traditional, AI-enhanced modeling, and hybrid computational strategies, this review outlines the progression of computational spine biomechanics toward patient-specific decision support and clinical integration.

## 2 Methods

### 2.1 Review design

This study was conducted as a structured narrative review aimed at conceptual synthesis of heterogeneous computational modeling methodologies rather than quantitative aggregation of clinical outcomes. The objective was to evaluate modeling strategies, validation approaches, and translational readiness across diverse computational spine modeling frameworks.

### 2.2 Literature search strategy

A structured literature search was conducted in PubMed, Scopus, and Web of Science for studies published between January 2010 and February 2026. Search queries included combinations of the following terms:

- “Lumbar spine”
- “Thoracolumbar”
- “Spinal deformity”
- “Finite element analysis”
- “Multibody modeling”
- “Musculoskeletal modeling”
- “Artificial intelligence”
- “Machine learning”
- “Physics-informed neural networks”

Broader spine-related terminology was included when studies contained lumbar biomechanical modeling components. Reference lists of relevant publications and prior reviews were additionally screened to identify eligible studies not captured in the primary search.

### 2.3 Eligibility criteria

Studies were included if they:

1. Employed finite element analysis (FEA), multibody (MB) musculoskeletal modeling, hybrid FE–MB frameworks, or AI-enhanced computational modeling of the lumbar spine.
2. Addressed degenerative, deformity-related, neoplastic, osteoporotic, or post-surgical lumbar spine conditions.
3. Reported model construction methodology and biomechanical outputs relevant to spinal mechanics or clinical application.

Peer-reviewed original research articles and technical modeling studies were considered.

Studies were excluded if they:

- Focused exclusively on non-lumbar spinal regions without lumbar biomechanical analysis.
- Lacked computational modeling components.
- Were purely clinical outcome studies without modeling methodology.

Validation status was not used as an exclusion criterion and was instead extracted for comparative evaluation of methodological maturity. No minimum model complexity requirements were imposed; model assumptions and completeness were analyzed comparatively due to heterogeneity of modeling approaches.

### 2.4 Data extraction and synthesis

For each included study, information was extracted regarding.

- Pathology modeled
- Modeling framework (traditional FEA, patient-specific FEA, AI-FEA, MB models, hybrid models)
- Geometric and material assumptions
- Boundary and loading conditions
- Validation approach (experimental, cadaveric, *in vivo*, retrospective clinical comparison)
- Stage of clinical translation

Due to substantial heterogeneity in modeling techniques, outcome metrics, and validation strategies, quantitative meta-analysis was not feasible. A qualitative thematic synthesis was performed. Studies were grouped by pathology and by modeling strategy to identify trends in methodological maturity, validation rigor, and translational readiness.

Common methodological limitations, including simplified loading assumptions, population-averaged material properties, incomplete disease-state modeling, and limited multicenter validation, were systematically cataloged to inform the analysis of translational barriers and future research directions.

## 3 Current finite element modeling approaches in lumbar spine pathology

Finite element analysis (FEA) has become a widely adopted computational tool for evaluating spinal biomechanics, including load distribution, stress patterns, and segmental range of motion under physiological and pathological conditions (Ahmadi et al., 2025b). In typical workflows, CT imaging is first segmented manually or semi-automatically to delineate cortical bone, cancellous bone, intervertebral discs, and posterior elements, after which surface geometries are converted into volumetric meshes and assigned nonlinear material properties based on published experimental data. Ahmadi et al., for example, described a streamlined but still operator-dependent pipeline in which patient CT data were segmented, smoothed, and meshed prior to application of physiologic compressive and bending loads (Ahmadi et al., 2025b). Their study demonstrated that even minor variations in segmentation boundaries and mesh refinement parameters altered predicted intradiscal pressure and facet joint forces, underscoring how operator decisions can influence mechanical outputs. Such findings highlight that model fidelity depends not only on parameter selection but also on the precision of geometric reconstruction methodology. Surface mesh optimization techniques, including Laplacian smoothing and geometric decimation, have improved model fidelity and numerical stability, allowing for more accurate representation of disc geometry and vertebral endplates (Ahmadi et al., 2025b). These methodological refinements enhance computational robustness but do not fully resolve variability in anatomical representation.

### 3.1 Model validation and biomechanical fidelity

Validation remains a critical requirement for biomechanical credibility. Across the literature, finite element predictions of range of motion, intradiscal pressure, and load sharing have been compared with cadaveric mechanical testing, *in vitro* measurements, *in vivo* experiments, and imaging-derived motion analyses (Sawa et al., 2020; Kiapour et al., 2022; Couvertier et al., 2017; Cai et al., 2020a; Doulgeris et al., 2023; Dreischarf et al., 2013; Mills and Sarigul-Klijn, 2019; Warren et al., 2020). Agreement across these experimental frameworks has supported the reliability of computational models for investigating spinal mechanics under controlled conditions. Collectively, these validation approaches demonstrate that model reliability depends on geometric fidelity and boundary condition selection rather than a single parameter choice.

Wiczenbach et al. developed and validated a modified FE model of the lumbar spine based on the Total Human Model for Safety (THUMS v6.1), incorporating orthotropic material properties and nonlinear stress-strain behavior for ligaments, nucleus pulposus, and annulus fibrosis (Wiczenbach et al., 2023). Their model was subjected to axial compression, shear loading, pure moments, and combined loading conditions, with outcomes including disc pressure and intervertebral rotation compared to cadaveric benchmarks. The agreement between computational and experimental data supported improved biomechanical fidelity (Wiczenbach et al., 2023).

Similarly, Stadelmann et al. demonstrated the predictive utility of homogenized FEA models in estimating the mechanical strength of metastatic vertebrae (Stadelmann et al., 2020). By accounting for three-dimensional bone mass distribution, FEA-based strength estimates outperformed conventional bone mineral content measurements. Notably, predictive accuracy was maintained across lytic, blastic, and mixed metastatic lesions without metastasis-specific material reparameterization (Stadelmann et al., 2020). Together, these studies illustrate that appropriately validated computational models can capture relevant mechanical behavior rather than purely theoretical mechanical trends.

### 3.2 Geometric variability and sensitivity

Despite successful validation in controlled settings, traditional FEA models often rely on fixed geometries and averaged material properties. This limitation constrains their ability to represent interpatient variability (Niemeyer et al., 2012). Niemeyer et al. performed a probabilistic uncertainty and sensitivity analysis of a fully parameterized, geometrically simplified model of the L3-L4 lumbar spine. They analyzed the impact of uncertainty on 40 geometric parameters. They found that the natural variability of the spine’s geometry strongly affects intradiscal pressure, range of motion, and facet joint contact forces (Niemeyer et al., 2012). These findings highlight the risk of extrapolating conclusions from single-geometry models to heterogeneous patient populations.

### 3.3 Probabilistic and population-based modeling

To address anatomical and material variability, probabilistic modeling frameworks such as Monte Carlo simulations have been introduced (Silvestros et al., 2019; Lee and Chung, 2022; Rijsbergen et al., 2018). These approaches systematically vary input parameters, including geometry, tissue properties, and loading conditions, across large simulated cohorts to quantify uncertainty and identify dominant biomechanical drivers (Silvestros et al., 2019; Lee and Chung, 2022). Niemeyer et al. performed a probabilistic sensitivity analysis of parameterized L3-L4 lumbar spine model in which 40 geometric variables were varied within physiologic ranges (Niemeyer et al., 2012). Their analysis demonstrated that small variations in endplate curvature, disc height, and facet orientation produced substantial changes in predicted intradiscal pressure and facet contact forces. Notably, geometric variability exerted greater influence on mechanical outputs than several material parameter perturbations, highlighting the potential limitations of single-geometry deterministic models (Niemeyer et al., 2012). These findings underscore the importance of accounting for interpatient variability when extrapolating computational results to heterogeneous clinical populations. Comparison with clinical and experimental measures suggests that probabilistic modeling improves characterization of inter-individual variability and uncertainty in biomechanical predictions (Silvestros et al., 2019).

Collectively, traditional and probabilistic FEA approaches provide critical biomechanical insight and validated mechanistic understanding. However, limitations in personalization, automation, and scalability have motivated the integration of artificial intelligence techniques to enhance efficiency and clinical applicability. Numerous studies have investigated biomechanical modeling across degenerative, structural, and neoplastic lumbar conditions using both traditional and emerging computational approaches. Representative examples across major pathologies are summarized in Table 1 (Vanaclocha et al., 2023; Wei et al., 2022; Ide et al., 2024; Pei et al., 2023; Zou et al., 2024; Xu et al., 2024; Saldarriaga et al., 2020; Hu et al., 2019; Park et al., 2025). Differences in validation depth and modeling assumptions across pathologies remain a major factor limiting direct clinical translation. These approaches shift modeling from single-representation analysis toward population-level inference, improving relevance to heterogeneous clinical cohorts.

**TABLE 1:** Maturity of biomechanical modeling approaches across major lumbar spine diseases.

Disease/pathology	FEA coverage in literature	AI-FEA integration	Validation status	Key gaps and limitations
Lumbar spinal stenosis	Extensive use of FEA to study decompression effects, load redistribution, and postoperative stability	Early integration for automated segmentation and patient-specific modeling	Moderate – cadaveric and small cohort validation studies	Limited dynamic loading and muscle force representation; unclear thresholds for surgical indication
Lumbar disc herniation	Well-established FEA models of annulus fibrosus, nucleus pulposus, and fiber-reinforced disc mechanics	Limited direct AI-FEA integration; AI mainly used for imaging and diagnosis	Moderate – validated against experimental and microstructural data	Limited translation to clinical prediction; complexity of disc microstructure modeling
Degenerative disc disease DDD	Extensive FEA studies examining stress redistribution, ROM changes, and adjacent segment degeneration	Emerging AI-FEA for alignment prediction and postoperative biomechanics	Moderate – retrospective and simulation-based validation	Incomplete disease-state modeling; limited prospective outcome correlation
Facet joint arthropathy	Moderate FEA coverage; often studied indirectly within DDD models	Limited AI-FEA application	Low – sparse experimental validation	Complex cartilage contact mechanics; inadequate representation of degeneration severity
Primary spinal tumors	Limited but growing FEA literature focused on vertebral strength and stability	Early AI-FEA exploration for risk stratification and elasticity estimation	Low to moderate – experimental and small-sample validation	Heterogeneous tumor properties; lack of large cohort studies
Metastatic spinal disease	Moderate FEA use for fracture risk and stabilization planning	Emerging AI-FEA for patient-specific failure prediction	Low to moderate – primarily *ex vivo* and retrospective validation	Limited integration of tumor progression and biological behavior
Scoliosis	Extensive FEA and MB modeling literature	Limited AI integration	Moderate validation (radiographic alignment + motion capture)	Long-term outcome prediction, disease progression modeling
Post-surgical lumbar spine fusion, arthroplasty	Extensive FEA evaluation of instrumentation and adjacent segment mechanics	Moderate AI-FEA for alignment and implant optimization	Moderate – retrospective and comparative validation	Limited long-term outcome prediction; high computational demands

Finite element analysis coverage, AI-FEA integration, validation status, and remaining gaps are summarized for degenerative, structural, and neoplastic lumbar pathologies, highlighting areas of translational readiness and priorities for future clinical validation. Degenerative disc disease and stenosis demonstrate extensive modeling coverage but limited large-cohort validation, whereas tumor-related applications remain comparatively underdeveloped. This heterogeneity in methodological maturity underscores the need for disease-specific validation strategies and improved translational standardization.

### 3.4 Musculoskeletal and multibody modeling

Musculoskeletal and multibody (MB) modeling approaches represent an alternative computational framework for investigating spinal biomechanics. Rather than resolving internal tissue stresses directly, these models estimate joint loading and muscle forces required to maintain posture and movement using rigid body mechanics and optimization algorithms. MB simulations enable evaluation of whole-body alignment, muscle activation patterns, and dynamic loading conditions that are difficult to capture using traditional finite element methods alone.

In spinal applications, multibody models have been used to study posture-dependent loading, compensatory muscle recruitment in deformity, and biomechanical consequences of altered sagittal balance. Musculoskeletal simulations demonstrate that representation of the multifidus and other paraspinal muscles substantially influences predicted lumbar joint loading, with simplified models tending to overestimate compressive forces (Wang K. et al., 2023). Whole-body musculoskeletal simulations further demonstrate that dynamic activities such as asymmetric lifting substantially increase lumbar shear forces and paraspinal muscle loading, highlighting the importance of activity-dependent modeling when evaluating injury mechanisms (Kim and Zhang, 2017). These approaches provide insight into global mechanical behavior and functional performance, complementing the tissue-level detail obtained from FEA. As a result, MB modeling is particularly useful for investigating activity-dependent biomechanics and patient-specific functional capacity.

However, because multibody approaches approximate anatomical structures as rigid segments, they cannot directly resolve internal tissue stress or localized failure patterns.

### 3.5 Coupled multibody-finite element frameworks

To overcome complimentary limitations of finite element and multibody approaches, coupled multibody-finite element (MB-FE) frameworks have been developed. In these models, muscle forces and joint reaction loads estimated from musculoskeletal simulations are applied as boundary conditions to finite element models enabling physiologically realistic loading while preserving tissue-level mechanical resolution. This integration allows simultaneous evaluation of global functional behavior and localized stress distributions, improving representation of posture-dependent biomechanics and activity-specific loading. Hybrid modeling has therefore emerged as a promising strategy for patient-specific analysis and surgical planning under physiologically realistic conditions.

Coupled musculoskeletal-finite element models further demonstrate that postoperative changes in adjacent segment loading depend on muscle-driven kinematics and lumbopelvic rhythm, highlighting the importance of physiologic loading conditions in predicting surgical biomechanical outcomes (Ebrahimkhani et al., 2022). Additionally, these hybrid simulations have demonstrated that surgical alterations to disc structure redistribute load towards facet joints, annulus tissue, and surrounding musculature; this provides a biomechanical explanation for postoperative pain recurrence (Haghighi et al., 2026). These developments increasingly enable simulation environments that approximate patient-specific mechanical behavior rather than idealized experimental conditions.

Collectively, these approaches illustrate a progression from isolated mechanical simulation toward integrated, functionally relevant representations of spinal biomechanics that better approximate clinical conditions.

## 4 Artificial intelligence-enhanced finite element modeling of lumbar spine diseases

**FIGURE 1:**
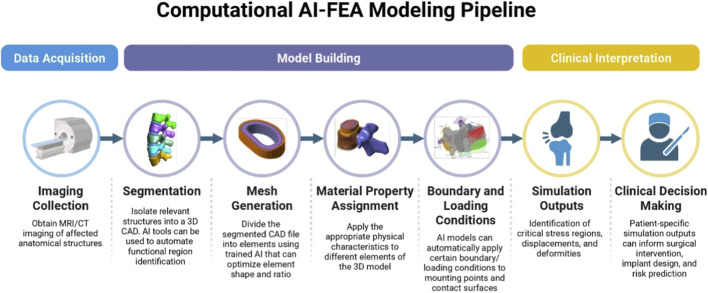
Computational AI-enhanced finite element modeling pipeline. Schematic representation of the artificial intelligence-enhanced finite element analysis (AI-FEA) workflow for patient specific biomechanical modeling. The process begins with imaging acquisition (MRI or CT), followed by automated or semi-automated segmentation to isolate relevant anatomical structures. The segmented data is converted into a finite element mesh, with AI-assisted optimization of element geometry and distribution. Material properties are then assigned to individual model components, incorporating tissue-specific characteristics. AI models may further assist in applying boundary and loading conditions based on anatomical landmarks and contact interfaces. Simulation outputs enable identification of stress distributions, displacements, and deformation patterns, which can subsequently inform clinical decision-making, including surgical planning, implant optimization, and risk prediction.

Artificial intelligence (AI) has increasingly influenced orthopedic and spinal research, supporting diagnostic workflows, perioperative planning and postoperative outcome prediction as depicted in Figure 1 (Wang and Wu, 2023; Han et al., 2025). Its adoption in lumbar spine disease management builds on successes in joint arthroplasty and orthopedic oncology, translating these methods into spine-specific workflows (Wang and Wu, 2023). In lumbar spine pathology, rather than replacing FEA, AI enhances each stage of the computational pipeline, from image segmentation to predictive modeling (Masharawi and Salame, 2011).

### 4.1 AI-driven automation of model generation

A primary contribution of AI to lumbar spine modeling lies in automated image segmentation. Deep learning algorithms enable rapid and reproducible identification of cortical and cancellous bone, intervertebral discs, ligaments, and cartilage from clinical imaging datasets (Ahmadi et al., 2025b). These segmented structures are converted into computational meshes and optimized using established geometric smoothing and decimation methods (Wiczenbach et al., 2023), substantially reducing operator dependence and processing time.

By standardizing model generation, AI facilitates consistent workflows across larger cohorts and mitigates limitations associated with manual segmentation. This standardization improves reproducibility and enables application across larger patient cohorts.

### 4.2 Patient-specific AI-FEA integration

Beyond segmentation, AI enables patient-specific FEA frameworks that incorporate individualized anatomy, degeneration patterns, and biomechanical loading (Masharawi and Salame, 2011). Variability in vertebral morphology (Näther et al., 2022), degeneration distribution (Ali et al., 2025) and load transmission (Gao et al., 2025; Phellan Aro et al., 2024) complicate surgical decision-making; AI-assisted FEA addresses these challenges by tailoring simulations to individual patients.

Patient-specific FEA has demonstrated improved predictive performance relative to generalized models by capturing subject-specific geometry and material properties (Shash and Elden, 2025). By integrating image-derived anatomy with individualized degeneration patterns and loading conditions, these frameworks enable more accurate estimation of stress distribution, implant loading, and alignment behavior. Such personalization supports applications in implant design, alignment optimization, and biomechanical risk assessment (Shash and Elden, 2025; Fereydoonpour et al., 2025), linking imaging-derived anatomy directly with patient-specific mechanical prediction to inform preoperative planning.

### 4.3 FEA-derived data for machine learning prediction

Beyond personalization of individual simulations, FEA also functions as a scalable data-generation engine for training predictive machine learning models. An emerging paradigm involves training machine learning models using FEA-generated datasets. Because clinical biomechanical datasets are limited in size and variability, physics-based simulations provide high-fidelity synthetic data suitable for supervised learning. This approach allows data-driven prediction while preserving mechanistic constraints from physics-based simulation.

For example, Phellan Aro et al. trained machine learning models on finite element-generated biomechanical simulations to predict postoperative spinal alignment following deformity correction (Phellan Aro et al., 2024). By generating large libraries of simulated alignment scenarios under varying geometric and loading conditions, their framework enabled rapid inference of sagittal alignment parameters without requiring repeated high-resolution FEA computation. Validation against postoperative radiographic measurements demonstrated high predictive accuracy, illustrating how simulation-derived datasets can bridge the gap between computational modeling and real-time clinical decision support.

In spinal oncology, CT-based finite element simulations have similarly been used to generate fracture risk datasets for metastatic vertebrae (Soltani et al., 2024; Garavelli et al., 2025). These models incorporate lesion size, location, and bone density distributions to estimate vertebral strength under physiologic loading. When combined with predictive modeling approaches, such frameworks support patient-specific fracture risk stratification and surgical planning.

Although predictive performance has been reinforced through validation against cadaveric datasets and CT-based mechanical assessments (Lerchl et al., 2024; Knapik et al., 2022), multicenter validation across large and heterogeneous patient populations remains limited (Saillard et al., 2024).

### 4.4 Clinical applications of AI-FEA in surgical planning

AI-enhanced FEA has expanded into preoperative planning and instrumentation optimization. AI-assisted systems can identify optimal pedicle screw trajectory and implant dimensions from imaging datasets, improving reproducibility and reducing breach risk (Gao et al., 2025; Phellan Aro et al., 2024). Simulation-based planning also enables evaluation of decompression extent and assessment of postoperative load redistribution. In metastatic disease, AI-augmented FEA supports vertebral strength estimation and stabilization decision-making (Chen et al., 2021). These applications suggest a shift from descriptive biomechanical modeling toward potential decision-support roles. Integration with visualization technologies such as virtual and augmented reality further enhances procedural rehearsal and intraoperative planning (Willaert et al., 2012; Caforio et al., 2025), enabling surgeons to evaluate biomechanical consequences before intervention.

### 4.5 Current challenges and future directions

Despite methodological advances, important limitations remain. Many AI-FEA frameworks rely on simplified static loading conditions and incompletely model dynamic muscle forces, reducing physiological realism (Willaert et al., 2012; Kalanjiyam et al., 2024). High-resolution patient-specific simulations may also impose substantial computational demands, limiting real-time implementation in resource-constrained settings (Fereydoonpour et al., 2025).

Furthermore, large-cohort external validation remains insufficient, constraining confidence in predictive generalizability (Saillard et al., 2024). Future development should emphasize hybrid modeling strategies integrating finite element methods with multibody dynamics to better represent muscle-driven loading and dynamic activity while maintaining computational efficiency (Zhang et al., 2025). Robust multicenter validation studies will be essential to establish reliability and accelerate clinical translation.

These limitations highlight the need for standardized validation and integration with physiologic loading models, topics addressed further in Section 6. A comparative overview of principal computational modeling frameworks discussed in this section is provided in Table 2.

**TABLE 2:** Comparison of computational modeling approaches applied to lumbar spine degeneration and tumors.

Approach	Primary inputs	Key strengths	Key limitations	Validation level in literature	Current clinical readiness
Traditional finite element analysis FEA	CT or MRI geometry, manual segmentation, population-averaged material properties, static or quasi-static loading	Established framework for stress, strain, ROM, and load distribution analysis; widely used in degeneration and tumor biomechanics	Generic anatomy and material properties; labor-intensive segmentation; simplified loading; limited patient specificity	Moderate – validated against cadaveric and experimental data in controlled settings	Research and preclinical planning
Deep learning–assisted FEA segmentation and meshing	CT or MRI, trained convolutional neural networks, automated mesh generation tools	Rapid, reproducible model preparation; reduced human error; enables scalable FEA workflows	Performance varies across anatomy and pathology; limited multicenter validation	Early to moderate – validated against manual segmentation in small cohorts	Enabling technology for research translation
Patient-specific FEA	Individual CT/MRI geometry, subject-specific material estimates, personalized boundary conditions	Captures interpatient variability; improves prediction of stress, failure risk, and postoperative biomechanics	Requires high-quality imaging and computational resources; validation still limited	Moderate – small cohort and retrospective validation studies	Early translational use
Probabilistic and Monte Carlo FEA	Distributions of geometry, material properties, and loading parameters	Quantifies uncertainty and population variability; improves robustness over deterministic models	Computationally intensive; indirect clinical interpretability	Moderate – validated against experimental variability	Research-focused
AI-augmented FEA machine learning trained on FEA libraries	Large-scale simulated FEA datasets, imaging features, clinical parameters	Real-time prediction of alignment and stress metrics; bridges limited clinical data gaps	Dependent on simulation assumptions; limited prospective validation	Early – primarily retrospective and proof-of-concept	Preclinical decision support
Physics-informed neural networks PINNs	Imaging-derived geometry, sparse biomechanical measurements, governing physical laws	Incorporates biomechanics directly into learning; reduces data requirements; rapid prediction	Limited clinical validation; complex implementation; disease-specific generalization not established	Early – technical and experimental validation	Research and early translational
Multibody MB musculoskeletal models	Rigid-body geometry, joint kinematics, muscle activation parameters	Captures muscle forces and whole-body dynamics; computationally efficient	Lacks detailed tissue-level stress and strain prediction	Moderate – validated against *in vivo* motion and muscle data	Research and biomechanical analysis
Coupled FEA–MB hybrid models	Combined FE tissue models and MB musculoskeletal dynamics	Integrates local tissue mechanics with global muscle-driven loading; improved physiological realism	High technical complexity; limited availability; computational burden	Early – limited *in vivo* validation	Experimental and research-stage

This comparison emphasizes that while Traditional FEA remains the most validated framework, AI-enhanced and hybrid approaches aim to improve scalability and personalization, though they remain in early translational stages. These distinctions are critical when evaluating claims of clinical applicability.

## 5 Clinical applications to lumbar spine diseases

The clinical translation of artificial intelligence-enhanced biomechanical modeling has expanded across lumbar spine degeneration and tumor-related instability. Rather than focusing on a single methodological framework, current applications encompass diagnostic assistance, surgical planning, implant optimization, and risk prediction. These approaches build upon conventional FEA while incorporating machine learning and emerging hybrid architectures to enhance scalability and personalization.

### 5.1 Spinal deformity and alignment modeling

Computational modeling has been applied to adult spinal deformity and scoliosis, where global alignment and load redistribution play central biomechanical roles. Finite element simulations have been used to evaluate curve progression, asymmetric disc loading, and the mechanical consequences of sagittal imbalance. Patient specific finite element modeling has also been applied to simulate scoliosis correction, with studies demonstrating close agreement between predicted and clinically observed postoperative alignment. Little and Adam demonstrated accurate prediction of surgical correction in adolescent scoliosis patients, supporting the role of patient-specific simulation in preoperative planning and validation of biomechanical models (Little and Adam, 2011). Altered spinal curvature modifies facet joint forces, ligament tension, and intervertebral disc stress distribution across multiple motion segments, supporting investigation of pain mechanisms and planning of corrective procedures such as osteotomy and fusion alignment strategies. Finite element studies incorporating thoracolumbar and pelvic alignment further demonstrate that both instrumentation configuration and correction technique influence complication risk, including rod fracture, proximal junctional kyphosis, and ligamentous injury during deformity correction (Imai et al., 2025; Kozaki et al., 2023). For example, Shekouhi et al. performed a finite element analysis evaluating pedicle subtraction osteotomy (PSO) at different lumbar levels and quantified the resulting changes in segmental stress distribution and global sagittal alignment (Shekouhi et al., 2024). Their simulations demonstrated that osteotomy level significantly influenced rod stress magnitude and adjacent segment loading, with lower lumbar PSO levels producing greater alignment correction but also higher implant stress concentrations. These findings provide a biomechanical explanation for rod fracture and proximal junction complications observed clinically, illustrating how finite element modeling can inform osteotomy level selection and instrumentation strategy during deformity correction.

### 5.2 Osteoporosis and vertebral fracture risk modeling

Computational modeling has also been widely applied to osteoporotic bone and vertebral fracture risk assessment. Finite element approaches allow estimation of vertebral strength and load-bearing capacity by incorporating bone mineral density distribution and trabecular architecture derived from CT imaging. These models have been used to evaluate fracture susceptibility under physiologic loading, assess the mechanical effects of vertebral augmentation procedures, and investigate failure mechanisms in weakened bone (Yan et al., 2022; Johannesdottir et al., 2021). CT-based finite element strength estimates have been shown to correlate with incident vertebral fractures, supporting their utility for identifying patients at elevated fracture risk beyond conventional bony density measures (Johannesdottir et al., 2021). Simulations further demonstrate that vertebral augmentation procedures can restore spinal stability and mechanical stiffness in osteoporotic compression fractures, supporting their role in treatment planning (Yan et al., 2022). Together with deformity and degenerative modeling, fracture-focused simulations illustrate how computational biomechanics can address both structural failure and progressive mechanical dysfunction across distinct spinal disease processes.

### 5.3 AI-assisted biomechanical characterizations in degeneration and tumor disease

Emerging AI-based modeling approaches have begun to characterize complex spinal biomechanics in clinically relevant contexts. Physics-informed neural networks (PINNs) represent one such emerging methodology that integrates governing physical laws directly into neural network training (Zhang et al., 2025). This improves model generalization and reduces dependence on large, labeled datasets.

Haghighat et al. demonstrated the application of PINNs for nonlinear constitutive model characterization, achieving parameter identification within minutes while maintaining errors below 10% (Haghighat et al., 2023). Similarly, Niu et al. developed a PINN framework capable of learning constitutive relationships from discrete stress-strain measurements while evaluating the influence of network architecture, inputs, and loss of function design (Niu et al., 2023). Building upon these foundational approaches, Zhang et al. introduced an enhanced PINN structure (EPINN-GF) integrating geometric features and physical constraints to improve stress prediction in complex spinal geometries (Zhang et al., 2025). Collectively, these studies illustrate a progression from material parameter estimation toward geometry-aware stress field prediction, expanding the potential for AI-driven biomechanical characterization in anatomically heterogeneous spinal conditions.

In the context of lumbar tumors, physics-informed modeling approaches have been explored for biomechanical characterization, elasticity estimation, and patient-specific fracture risk (Kamali et al., 2023). By combining imaging data with embedded mechanical laws, these models can estimate spatial distributions of mechanical properties within heterogeneous tumor-involved vertebrae. This facilitates assessment of erosion extent, structural instability, and load-bearing capacity, which are clinically relevant for surgical planning and implant selection (Wiczenbach et al., 2023). However, these applications remain largely methodological or proof-of-concept, and broad clinical validation has not yet been established.

### 5.4 Clinical deployment of AI-Based diagnostic and predictive models

Conventional deep learning and machine learning models currently demonstrate greater clinical maturity. Diagnostic tools such as SpineNet have shown agreement rates with radiologists ranging from 78% to 92% in grading lumbar disc degeneration and related pathologies (McSweeney et al., 2023). These systems assist in standardizing imaging interpretation and reducing interobserver variability. Machine learning approaches have also been applied to preoperative risk stratification, implant selection, and prediction of postoperative complications, with reported predictive accuracies approaching 92% in selected cohorts (Karandikar et al., 2022). Such tools enhance visualization, supporting personalized surgical strategies, whereas PINN-based frameworks remain in early developmental strategies.

### 5.5 Clinical decision support and personalized treatment planning

Computational spine modeling increasingly functions as a clinical decision-support tool. By integrating imaging data, patient-specific anatomy, and predicted mechanical response, simulation frameworks can assist surgeons in evaluating treatment strategies prior to intervention.

In deformity correction, modeling has been used to compare alignment targets and estimate mechanical consequences of alternative correction strategies and construct configurations. For degenerative diseases, patient-specific simulations can evaluate adjacent segment loading, implant positioning, and decompression extent to minimize postoperative instability. Comparisons of rigid and flexible fixation constructs further show that implant selection alters adjacent segment stresses and motion, supporting the use of biomechanical simulation for preoperative construct optimization (Khalaf and Nikkhoo, 2021).

**FIGURE 2:**
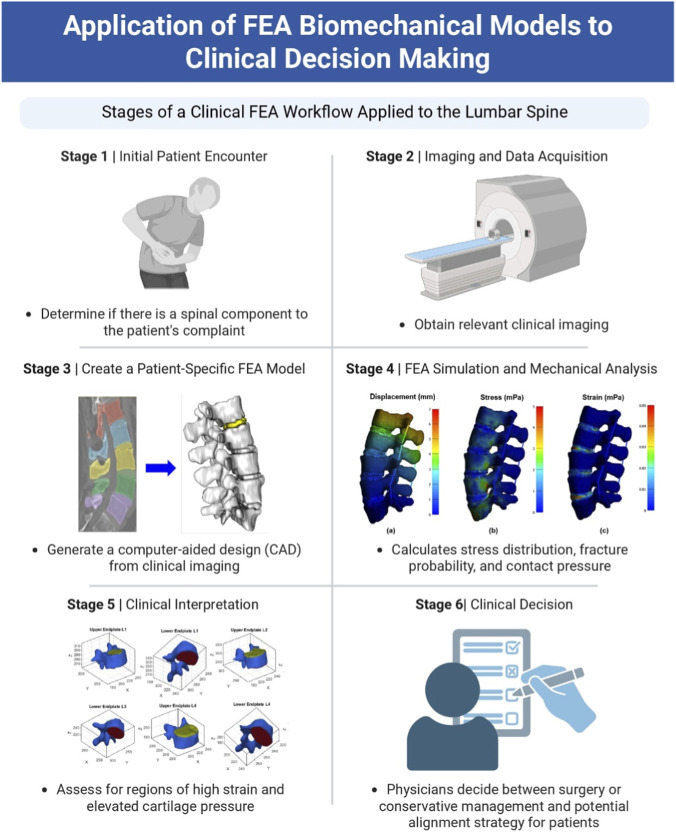
Integration of finite element modeling into clinical decision-making workflows. Conceptual representation of how FEA can be incorporated into staged clinical care pathways for the lumbar spine. Following initial patient evaluation and imaging acquisition such as CT or MRI of the lumbar region, patient-specific models of vertebral bodies and intervertebral disks are generated from clinical datasets and subjected to biomechanical simulation. Mechanical outputs, including vertebral stress distributions, disc strain patterns, endplate loading, fracture risk, and facet joint contact pressures, are interpreted within the clinical context to inform management decisions. Applications extend across diverse pathologies, including degenerative disc disease, arthritis, disc herniation, spinal stenosis, aneurysms, vertebral compression fractures, spondylolisthesis, tumors, and degenerative or structural disorders. This framework illustrates the progression of computational biomechanics in the lumber spine from investigational modeling towards structured decision-support integration.

Similarly, in tumor-related instability and fracture risk assessment, computational models can help determine the need for prophylactic stabilization and guide instrumentation planning. Finite element analyses of metastatic vertebrae demonstrate prophylactic stabilization procedures can improve vertebral strength while limiting excessive adjacent-level stress, supporting simulation-based decision-making in oncologic spine management (Berton et al., 2020). Collectively, these applications illustrate the transition of computational modeling from retrospective biomechanical analysis toward prospective, patient-specific treatment planning. The staged integration of finite element modeling in clinical decision-making workflows is illustrated in Figure 2.

In addition to preoperative planning, computational modeling may also support postoperative monitoring and outcome assessment. Patient-specific simulations can be updated using follow-up imaging to evaluate evolving load distribution, implant stresses, and adjacent segment mechanics after surgery. Integration with longitudinal imaging and clinical data may allow for early identification of mechanical complications such as implant loosening, progressive deformity, or adjacent segment degeneration. In this context, modeling frameworks function not only as planning tools but as dynamic assessment platforms capable of informing postoperative management and rehabilitation strategies.

### 5.6 Translational challenges and future clinical integration

Although these applications demonstrate clinical potential, widespread adoption remains limited and depends on the validation and standardization considerations discussed in Section 6.

Key translational barriers include lack of standardized validation protocols, limited multicenter clinical datasets, regulatory considerations, and insufficient longitudinal outcome reporting. Bridging this gap will require prospective validation studies comparing AI-FEA and PINN-based approaches with established surgical planning workflows. As validation expands, hybrid modeling integrating mechanistic simulation and machine learning may offer a pathway toward clinically deployable patient-specific decision support systems. To contextualize computational modeling within real-world clinical workflows, Table 3 outlines representative clinical use cases of finite element and AI-enhanced modeling strategies, including their evidentiary basis and current stage of translational adoption.

**TABLE 3:** Clinical applications of finite element and AI-enhanced modeling in lumbar spine disease.

Disease category	Clinical application	Modeling method used	Primary evidence type	Status	Key references
Degenerative conditions	Preoperative decompression planning for lumbar stenosis	Traditional FEA; patient-specific FEA	Retrospective simulation studies; cadaveric validation	Preclinical decision support	Ahmadi et al. (2025a), Ahmadi et al. (2025b), Kiapour et al. (2022)
	Adjacent segment degeneration risk assessment	FEA; probabilistic FEA	Simulation studies; sensitivity analyses	Research-stage	Niemeyer et al. (2012), Rijsbergen et al. (2018), Ebrahimkhani et al. (2022)
	Interbody cage design and selection	Patient-specific FEA; AI-driven FEA optimization	Simulation studies; comparative biomechanical analyses	Preclinical and translational	Vanaclocha et al. (2023), Zou et al. (2024), Xu et al. (2024), Kim and Zhang (2017)
	Postoperative outcome and complication prediction	Machine learning with FEA-derived features	Retrospective cohort studies	Early translational	Phellan Aro et al. (2024), McSweeney et al. (2023)
Spinal deformity	Prediction of postoperative alignment and range of motion	AI-augmented FEA; ML trained on FEA libraries	Retrospective cohort analyses; simulation-based validation	Early translational	Phellan Aro et al. (2024), Kozaki et al. (2023)
	Virtual surgical rehearsal and scenario testing	AI-FEA integrated with VR/AR visualization	Technical feasibility studies	Early clinical adoption	Willaert et al. (2012), Caforio et al. (2025)
Osteoporosis/Vertebral fracture risk	Vertebral fracture risk estimation	FEA; AI-FEA risk stratification models	Experimental testing; retrospective modeling	Research and early translational	Imai et al. (2025), Soltani et al. (2024), Garavelli et al. (2025), Shekouhi et al. (2024)
Tumor related instability	Tumor related vertebral fracture risk estimation	FEA; AI-FEA risk stratification models	Experimental testing; retrospective modeling	Research and early translational	Lai et al. (2025), Stadelmann et al. (2020), Soltani et al. (2024), Khalaf and Nikkhoo (2021)
	Stabilization strategy selection in spinal tumors	FEA of instrumentation constructs	Simulation and cadaveric studies	Research-stage	Park et al. (2024), Khalaf and Nikkhoo (2021)
Instrumentation and implant optimization	Pedicle screw trajectory and sizing optimization	AI-assisted surgical planning with FEA validation	Retrospective technical validation	Early clinical adoption	Gao et al. (2025), Phellan Aro et al. (2024)
	Material and implant design optimization	AI-driven FEA	Simulation-based optimization	Preclinical	Vanaclocha et al. (2023), Shash and Elden (2025)

Representative applications of traditional FEA, AI-FEA, MB modeling, and machine learning approaches organized by disease category. Foreach application, the predominant modeling framework, primary type of supporting evidence, translational status, and key supporting references are provided. Applications are categorized into degenerative conditions, spinal deformity, osteoporosis/fracture risk, tumor-related instability, and cross-pathology instrumentation or implant optimization to clarify clinical alignment and evidentiary maturity.

Across clinical applications, computational modeling demonstrates variable levels of translational maturity. Traditional finite element studies primarily provide mechanistic interpretation of pathology, whereas AI-enhanced and hybrid frameworks increasingly target prediction and decision support. Deformity and fracture applications show the greatest proximity to clinical workflow integration due to direct mechanical outcome relationships, while degeneration and pain-related conditions remain more complex due to multifactorial symptom generation. Consequently, the progression from biomechanical analysis to clinical implementation appears to depend less on model sophistication and more on the strength of the link between mechanical metrics and measurable clinical outcomes. This distinction highlights why certain spinal conditions are more amenable to modeling-guided decision-making than others. Evidence from applications in orthopedics, oncology, and deformity correction demonstrates that finite element modeling can achieve meaningful clinical integration when strong relationships exist between mechanical predictions and measurable clinical outcomes. These observations support the potential for similar translational progress in lumbar spine disease, provided that validation frameworks and outcome-based evidence are appropriately developed.

## 6 Gaps and future directions for clinical translation

Despite advances in finite element analysis (FEA), multibody (MB) modeling, and AI-enhanced computation frameworks, routine clinical implementation in lumbar spine degeneration and tumor instability remains limited. Translational barriers relate primarily to validation, personalization, standardization, and integration into clinical workflows rather than fundamental biomechanical feasibility.

### 6.1 Validation and standardization challenges

FEA and MB models are well-established computational tools for evaluating spinal biomechanics (Lisiak-Myszke et al., 2020). In selected contexts, these approaches have demonstrated potential to inform clinical decision-making (Wei et al., 2022; Christen et al., 2010; Carpenedo et al., 2025). However, most current models rely on generalized anatomical geometries and averaged material properties that may not accurately represent disease-specific or patient-specific variations (Ahmadi et al., 2025b; Ahmadi et al., 2025c). Furthermore, simplified boundary and loading conditions, often excluding physiologic muscle forces, may influence predicted stress distributions and segmental motion (Pfeiffer, 2016).

**FIGURE 3:**
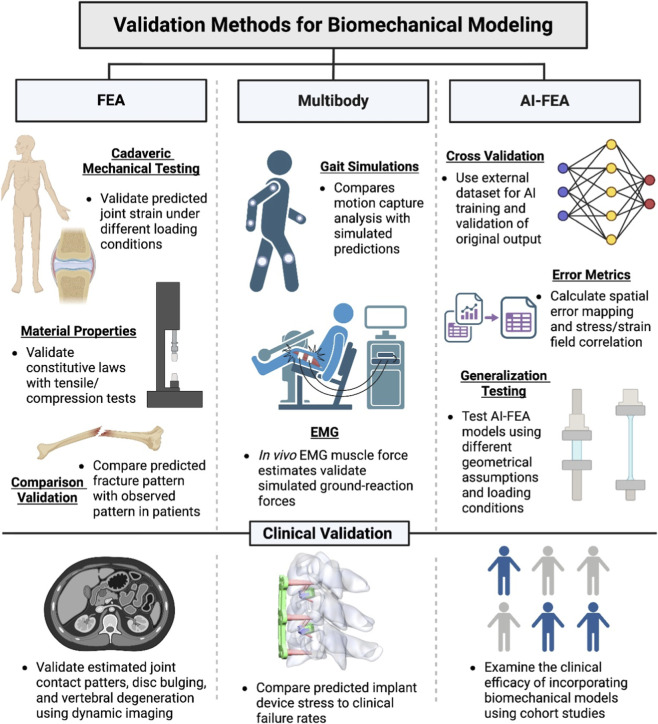
Validation Methods for biomechanical Modeling Frameworks. Overview of validation strategies across FEA, MB, musculoskeletal modeling, and AI-FEA frameworks. Traditional FEA validation includes cadaveric mechanical testing, experimental material property characterization, and comparison of predicted fracture patterns or stress distributions with clinical observations. Multibody modeling validation commonly incorporates gait simulations, motion capture analysis, and electromyography (EMG)-derived muscle force comparisons to assess physiologic loading accuracy. AI-FEA validation requires additional cross-validation using external databases, spatial error mapping of predicted stress/strain fields, and generalization testing across variable geometric and loading conditions. Future validation research should use clinical validation methods like dynamic imaging, clinical failure rates of lumbar spine procedures, and large randomized-controlled trials comparing outcomes in FEA-informed surgical approaches to traditional approaches. These methods can be used to advance biomechanical model integration in routine clinical practice.

Although automated segmentation and AI-assisted modeling techniques have improved efficiency (Bayoglu et al., 2023), prospective validation against clinical outcomes remains sparse (Stops et al., 2012). Most validation studies focus on mechanical outputs rather than demonstrating improvement in patient-centered outcomes. Establishing standardized reporting frameworks for model assumptions, boundary conditions, and validation benchmarks is essential to ensure reproducibility and comparability across studies. Representative validation strategies across traditional finite element, multibody, and AI-enhanced modeling frameworks are summarized in Figure 3.

In addition to commonly reported validation approaches based on cadaveric testing or generalized mechanical benchmarks, direct validation using patient-specific data remains limited. Comparisons between personalized model predictions and *in vivo* measurements, such as segmental kinematics derived from imaging or intradiscal pressure measurements, represent a critical step toward establishing clinical credibility (Sawa et al., 2020; Kiapour et al., 2022; Couvertier et al., 2017). However, such approaches are infrequently implemented due to challenges in acquiring high-resolution, patient-specific biomechanical data.

Furthermore, validation frameworks incorporating pre- and postoperative comparisons remain underdeveloped. Studies that evaluate changes in alignment, load distribution, or mechanical behavior before and after surgical intervention provide an opportunity to assess the predictive utility of computational models in clinically relevant scenarios (Phellan Aro et al., 2024). Cases involving coronal deformities introduce three-dimensional complexity that extends beyond simplified or planar modeling assumptions. Incorporating these more complex clinical conditions into validation strategies is essential for assessing model robustness and improving translational applicability.

### 6.2 Personalization and scalability of FEA frameworks

The integration of patient-specific imaging data into predictive FEA pipelines remains technically challenging (Ahmadi et al., 2025b; Ahmadi et al., 2025c). Automation strategies such as AI-driven segmentation have significantly reduced model generation time (Bayoglu et al., 2023), yet scalability to large and heterogeneous patient cohorts has not been achieved in routine practice. Current workflows often require substantial preprocessing, manual quality control, and computational resources that limit the number of cases that can be processed efficiently in time-sensitive clinical environments.

Hybrid AI-FEA frameworks incorporating neural networks for material property estimation and geometric adaptation demonstrate promising predictive accuracy (Remus et al., 2023), but robust external validation across diverse patient populations is lacking (Stops et al., 2012). Many reported performance metrics are derived from retrospective or single-center datasets, raising uncertainty regarding generalizability to broader clinical settings.

Similarly, retrospective computational approaches have demonstrated the ability to predict postoperative spinopelvic alignment with accuracies ranging from 81% to 94% (Stops et al., 2012). Although not based on full FEA modeling, such validation paradigms illustrate the type of large-cohort, outcome-linked evaluation necessary for broader translational adoption. Future progress will depend not only on improving personalization algorithms but also on demonstrating reproducible clinical benefit across institutions and patient populations.

An additional limitation in current computational modeling frameworks is the limited incorporation of sex- and age-specific biological variation. Many models rely on generalized or population-averaged material properties, with personalization often restricted to geometric scaling or simplified adjustments such as body weight or estimated muscle strength. However, sex- and age-related differences in bone density, disc composition, ligament stiffness, and muscle function can substantially influence spinal biomechanics and load distribution. For example, age-related changes in intervertebral disc hydration and bone mineral density, as well as sex-specific differences in vertebral morphology and muscle mass, may alter both mechanical response and failure patterns (Inoue and Espinoza Orías, 2011; Yan et al., 2022; Masharawi and Salame, 2011). Incorporating sex-, age-, and disease-specific biological variation will be critical for improving biological realism, enhancing predictive accuracy, and advancing clinical applicability of computational spine models.

### 6.3 Coupling finite element and multibody models

MB models and FEA differ fundamentally in their representation of spinal mechanics. MB approaches typically model vertebrae as rigid bodies connected by joints, with muscles represented as force-generating elements, enabling simulation of whole-body dynamics and muscle recruitment patterns (Bayoglu et al., 2023). In contrast, FEA resolves detailed tissue-level stresses, strains, and contact interactions with discs, ligaments, and facet joints.

Hybrid MB-FE models integrate muscle-driven dynamics with structural tissue mechanics (Ghezelbash et al., 2025). Both unidirectional approaches, where muscle forces derived from MB simulations are applied to FE models, and bidirectional frameworks allowing mechanical feedback between tissue deformation and musculoskeletal systems have been described. Remus et al. developed and validated a hybrid lumbosacral spine model within the ArtiSynth platform, combining multibody dynamics with finite element representations of passive spinal structures and validating model outputs against *in vivo* intradiscal pressure and muscle activity data (Remus et al., 2021). Such frameworks demonstrate that physiologically realistic loading patterns can be reproduced while preserving tissue-level mechanical fidelity.

**FIGURE 4:**
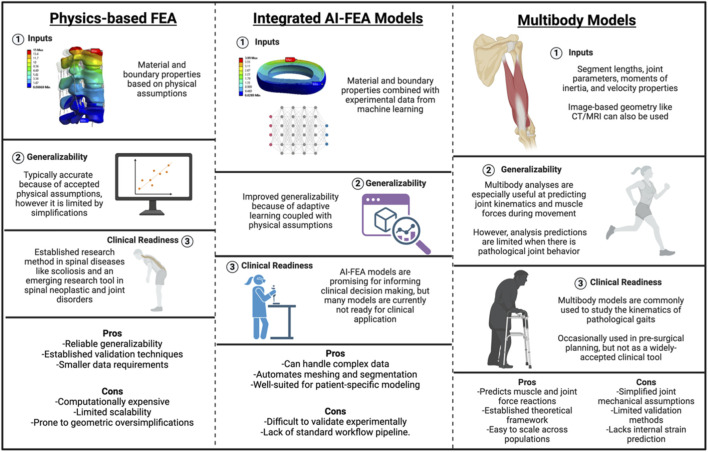
Comparative overview of biomechanical modeling frameworks. Conceptual comparison of physics-based FEA, AI-FEA, and MB musculoskeletal modeling approaches. Traditional physics-based FEA relies on experimentally derived material properties and boundary conditions grounded in mechanical theory, offering established validation pathways but limited scalability. AI-integrated FEA combines data-driven learning with physical constraints to improve automation, personalization, and generalizability, though validation and workflow standardization remain evolving. Multibody models emphasize joint kinematics and muscle force prediction, providing efficient population-level analysis but lacking detailed internal stress and strain resolution. The relative advantages and limitations of each framework underscore the complementary roles of these approaches in advancing translational computational spine modeling.

To improve computational efficiency, surrogate modeling techniques have also been introduced. Hammer et al. developed energy-conserving polynomial surrogate models of intervertebral disc mechanics embedded within multibody simulations (Hammer et al., 2024). These approaches preserve essential nonlinear disc behavior while substantially reducing computational cost, enabling large-scale musculoskeletal analyses without repeat high-resolution FE recalculation. Despite these advances, coupled MB-FE frameworks remain technically challenging due to differences in mathematical formulation, numerical integration schemes, and stability constraints. Large-cohort validation and standardized benchmarking remain limited (Li et al., 2019; Campbell et al., 2020). In addition to these technical challenges, current coupled MB-FE approaches are limited by the lack of validated methods for predictive physiologic muscle activation patterns. Most models rely on optimization-based or inverse dynamics strategies to estimate muscle forces (Wang K. et al., 2023; Kim and Zhang, 2017), which may not accurately reflect true neuromuscular control or patient-specific activation behavior. A conceptual comparison of physics-based finite element analysis, integrated AI-enhanced FEA, and multibody modeling frameworks is summarized in Figure 4.

An additional and underemphasized limitation in current computational spine modeling is the lack of predictive, active simulations that integrate patient-specific anatomy with dynamically generated muscle forces and time-dependent motion. Existing approaches that incorporate musculature often rely on simplified assumptions, such as constant or predefined muscle forces in finite element models, or inverse dynamics frameworks based on recorded motion data in multibody simulations (Wang K. et al., 2023; Kim and Zhang, 2017). While these methods provide valuable insight into spinal loading under specific conditions, they are not inherently predictive and cannot fully capture how patients may respond to novel interventions or changing biomechanical environments.

The integration of active motion simulation, patient-specific modeling, and forward predictive frameworks remains underdeveloped, particularly when coupled with appropriate dynamic validation datasets. Most current validation strategies focus on quasi-static or statistical comparisons (Bayoglu et al., 2023), which may not adequately reflect time-dependent biomechanical behavior. However, dynamic simulations are likely essential for evaluating clinically relevant outcomes such as implant fatigue, failure risk, and the progression of adjacent segment degeneration. Advancing toward predictive, dynamically validated modeling frameworks represents a key direction for future research and a necessary step for meaningful clinical translation.

### 6.4 Disease-specific modeling limitations

Certain pathologies pose unique modeling challenges. Facet joint arthropathy, for example, involves complex cartilage geometry, variable thickness distributions, and evolving contact mechanics. Many existing FEA models simplify facet cartilage behavior and contact interactions (Woldtvedt et al., 2011; Cai et al., 2020b). This potentially limits predictive accuracy in degenerative conditions. A systematic review reported that only a minority of facet joint FEA studies included experimental validation of outputs (Woldtvedt et al., 2011).

Additionally, degenerative alterations in facet orientation, tropism, and load-sharing interactions further complicate accurate modeling (Park et al., 2024; Bashkuev et al., 2020). The limited availability of experimental data characterizing advanced facet joint degeneration constrains validation efforts (Kalanjiyam et al., 2024; Du et al., 2016). Addressing these disease-specific modeling gaps through improved experimental characterization and standardized validation strategies will be critical for enhancing reliability and clinical confidence (Ahuja et al., 2020).

### 6.5 Interpretation of modeling outputs in clinical context

Quantitative outputs from computational models represent the mechanical environment of the spine rather than direct measures of symptoms or clinical outcomes. Pain generation, neurological compromise, and functional impairment depend on multifactorial biological processes including inflammation, neural sensitization, and patient-specific tolerance to mechanical change. Consequently, biomechanical predictions should be interpreted as indicators of mechanical conditions rather than deterministic predictors of patient experience.

In clinical use, simulation results may therefore be most informative when applied comparatively rather than absolutely. Evaluating relative differences between treatment strategies, alignment targets, or implant configurations is typically more reliable than attempting to predict specific postoperative symptoms. Establishing clinically meaningful thresholds for mechanical parameters will require prospective correlation studies, and the absence of such thresholds currently limits decision-making based solely on simulation results.

### 6.6 Pathway toward clinical implementation

For FEA, MB, and AI-enhanced hybrid models to transition into routine clinical practice, several criteria must be met:

1. Prospective, multicenter validation linking model predictions to clinical decision-making and patient outcomes.
2. Standardized reporting of model assumptions, boundary conditions, and validation metrics.
3. Robust patient-specific modeling pipelines integrating imaging, material characterization, and physiologic loading.
4. Demonstration that AI-enhanced or coupled models provide measurable improvements over existing planning strategies.

Collectively, Tables 1–3 demonstrate that while methodological sophistication has advanced across finite element, AI-enhanced, and hybrid modeling paradigms, validation depth and clinical integration remain heterogeneous across pathologies. Addressing these requirements will determine whether computational spine modeling evolves from a powerful research methodology into a clinically reliable decision-support framework.

Collectively, these limitations indicate that the primary barrier to clinical adoption is no longer computational capability but standardization and evidence generation. Future progress will depend on demonstrating reproducible improvements in clinical decision-making rather than solely improving biomechanical fidelity. In this context, computational modeling should be interpreted as a supplementary decision-support tool analogous to advanced imaging biomarkers, requiring clinical correlation and standardized interpretation guidelines. Establishing shared benchmarks and outcome-linked validation strategies will therefore represent a critical step in transitioning computational spine modeling from investigational methodology to routine clinical infrastructure.

## 7 Conclusion

Computational modeling has become an important tool for understanding the biomechanical consequences of lumbar spine disease, including degenerative, deformity, osteoporotic, and neoplastic conditions. Finite element analysis provides mechanistic insight into load transmission, segmental motion, and tissue-level stress patterns that are not directly measurable *in vivo*, while multibody and hybrid frameworks extend these capabilities to functional and muscle-driven loading environments. Integration of artificial intelligence has accelerated model generation, enabled patient-specific parameter estimation, and expanded predictive simulation strategies. Together, these developments shift computational biomechanics from isolated mechanical analysis toward clinically oriented simulation capable of informing surgical planning, risk assessment, and postoperative monitoring. Despite substantial methodological progress, the principal barrier to clinical adoption remains validation rather than computational feasibility. Multicenter outcome-based evaluation, standardized reporting of model assumptions, and demonstration of reproducible clinical benefit will be required before computational models can reliably guide routine decision-making. Consequently, current modeling approaches should be considered adjunctive tools that complement clinical judgment rather than replace it. Continued emphasis on reproducibility, transparency, and prospective clinical evidence will determine whether computational spine modeling evolves from an investigational methodology into an integrated component of precision musculoskeletal care.
